# A Structural Model of the *Staphylococcus aureus* ClfA–Fibrinogen Interaction Opens New Avenues for the Design of Anti-Staphylococcal Therapeutics

**DOI:** 10.1371/journal.ppat.1000226

**Published:** 2008-11-28

**Authors:** Vannakambadi K. Ganesh, Jose J. Rivera, Emanuel Smeds, Ya-Ping Ko, M. Gabriela Bowden, Elisabeth R. Wann, Shivasankarappa Gurusiddappa, J. Ross Fitzgerald, Magnus Höök

**Affiliations:** 1 Center for Extracellular Matrix Biology, Institute of Biosciences and Technology, Texas A & M University Health Science Center, Houston, Texas, United States of America; 2 Centre for Infectious Diseases, School of Biomedical Sciences, The University of Edinburgh, Edinburgh, United Kingdom; The University of Texas-Houston Medical School, United States of America

## Abstract

The fibrinogen (Fg) binding MSCRAMM Clumping factor A (ClfA) from *Staphylococcus aureus* interacts with the C-terminal region of the fibrinogen (Fg) γ-chain. ClfA is the major virulence factor responsible for the observed clumping of *S. aureus* in blood plasma and has been implicated as a virulence factor in a mouse model of septic arthritis and in rabbit and rat models of infective endocarditis. We report here a high-resolution crystal structure of the ClfA ligand binding segment in complex with a synthetic peptide mimicking the binding site in Fg. The residues in Fg required for binding to ClfA are identified from this structure and from complementing biochemical studies. Furthermore, the platelet integrin α_IIb_β_3_ and ClfA bind to the same segment in the Fg γ-chain but the two cellular binding proteins recognize different residues in the common targeted Fg segment. Based on these differences, we have identified peptides that selectively antagonize the ClfA-Fg interaction. The ClfA-Fg binding mechanism is a variant of the “Dock, Lock and Latch” mechanism previously described for the *Staphylococcus epidermidis* SdrG–Fg interaction. The structural insights gained from analyzing the ClfANFg peptide complex and identifications of peptides that selectively recognize ClfA but not α_IIb_β_3_ may allow the design of novel anti-staphylococcal agents. Our results also suggest that different MSCRAMMs with similar structural organization may have originated from a common ancestor but have evolved to accommodate specific ligand structures.

## Author Summary

*Staphylococcus aureus* (*S. aureus*) is a common pathogen that can cause a range of diseases from mild skin infections to life-threatening sepsis in humans. Some surface proteins on *S. aureus* play important roles in the *S. aureus* disease process. One of these bacterial surface proteins is clumping factor A (ClfA) that binds to the C-terminal region of one of the three chains of fibrinogen (Fg), a blood protein that plays a key role in coagulation. We carried out biochemical and structural studies to understand the binding mechanism of ClfA to Fg and to define the residues in Fg that interact with ClfA. Interestingly, the platelet integrin, which is important for platelet aggregation and thrombi formation, also binds to the same region of Fg as ClfA. Despite the fact that the two proteins bind at the same region, the mode of recognition is significantly different. Exploiting this difference in recognition, we have demonstrated that agents could be designed that inhibit the ClfA–Fg interaction but do not interfere with the interaction of Fg with the platelet integrin. This opens the field for the design of a novel class of anti-staph therapeutics.

## Introduction

*Staphylococcus aureus* is a Gram-positive commensal organism that permanently colonizes 20% of healthy adults and transiently colonizes up to 50% of the general population [1]. For many years, *S. aureus* has been a major nosocomial pathogen causing a range of diseases from superficial skin infections to life-threatening conditions, including septicemia, endocarditis and pneumonia [1],[2]. Within the last decade a dramatic increase in the number of invasive infections caused by community-acquired *S. aureus* have been recorded in otherwise healthy children and young adults [3],[4]. This outbreak together with the continued increase in antibiotic resistance among clinical strains underscores the need for new prevention and treatment strategies [1].

A detailed characterization of the molecular pathogenesis of *S. aureus* infections may expose new targets for the development of novel therapeutics. Several staphylococcal virulence factors have been identified including capsule, surface adhesins, proteases, and toxins (reviewed in [5],[6],[7],[8]). One of these virulence factors is the MSCRAMM (microbial surface components recognizing adhesive matrix molecules) clumping factor A (ClfA). ClfA is the major staphylococcal fibrinogen (Fg) binding protein and is responsible for the observed clumping of *S. aureus* in blood plasma [9],[10]. Essentially all *S. aureus* clinical strains carry the *clfA* gene [11]; ClfA is a virulence factor in a mouse model of septic arthritis [12] and in rabbit and rat models of infective endocarditis [13],[14],[15].

ClfA generates strong immune responses and has shown potential as a vaccine component in active and passive immunization studies. In one study, mice vaccinated with a recombinant ClfA segment containing the Fg-binding domain and subsequently challenged with *S. aureus* showed significantly lower levels of arthritis compared to mice vaccinated with a control protein [12]. In another study, mice passively immunized with polyclonal or monoclonal antibodies against the ClfA Fg-binding domain were protected in a model of septic death [16]. The humanized monoclonal antibody, Aurexis®, has a high affinity for ClfA and inhibits ClfA binding to Fg [17]. Aurexis is currently in clinical trials in combination with antibiotic therapy for the treatment of *S. aureus* bacteremia [18]. Thus ClfA is a viable target for both vaccine and therapeutic strategies.

ClfA belongs to a class of cell wall-localized proteins that are covalently anchored to the peptidoglycan [5],[19],[20]. Starting from the N-terminus, ClfA contains a signal sequence followed by the ligand-binding A region composed of three domains (N1, N2, and N3), the serine-aspartate repeat domain (R region), and C-terminal features required for cell wall anchoring such as the LPXTG motif, a transmembrane segment and a short cytoplasmic domain [21],[22],[23]. A crystal structure of a Fg-binding ClfA segment (residues 221–559) which includes two of the domains (N2N3) demonstrates that each domain adopts an IgG-like fold [24]. This domain architecture was also determined from the crystal structure of the ligand binding segment of SdrG from *Staphylococcus epidermidis*, an MSCRAMM that binds to the N-terminal region of the Fg β-chain [25].

A dynamic mechanism of Fg binding termed “Dock, Lock and Latch” (DLL) has been proposed for SdrG based on a comparison of the crystal structures of SdrG N2N3 as an apo-protein and in complex with a synthetic peptide mimicking the targeted site in Fg [25]. In the SdrG DLL model, the apo-form of the protein adopts an open conformation that allows the Fg ligand access to a binding trench between the N2 and N3 domains. As the ligand peptide docks into the trench, a flexible C-terminal extension of the N3 domain is redirected to cover the ligand peptide and “lock” it in place. Subsequently the C-terminal part of this extension interacts with the N2 domain and forms a β-strand complementing a β-sheet in the N2 domain. This inserted β-strand serves as a latch to form a stable MSCRAMM ligand complex.

ClfA binds to the C-terminus of the Fg γ-chain [10],[23] and a synthetic 17 amino acid peptide corresponding to this region was shown to bind to ClfA. Interestingly, the A-region of the staphylocccal MSCRAMM FnbpA protein also binds to the same region in Fg [23]. Moreover residues in this Fg segment are also targeted by the platelet α_IIb_β_3_ integrin [26],[27],[28] and a recombinant form of ClfA has been shown to inhibit platelet aggregation and the binding of platelets to immobilized Fg [10],[29],[30].

The current study was undertaken to characterize the interaction of ClfA and Fg to define in detail the binding of the C-terminus of Fg's γ-chain and to explore if compounds can be constructed that antagonize the ClfA-Fg interaction but does not affect the Fg interaction with the platelet-integrin α_IIb_β_3_.

## Results/Discussion

### Identification of critical residues in Fg required for binding to ClfA

**Figure 1:**
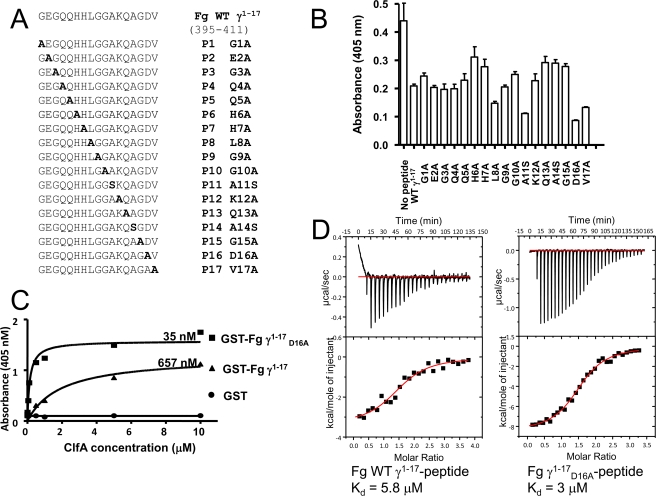
ClfA_229–545_ binds to Fg γ chain peptides. (A) Panel of Fg γ-chain peptides. The wild-type peptide corresponds to the 17 C-terminal residues of the Fg γ-chain (395–411); the mutated peptides have individual amino acids replaced with Ala (or Ser). (B) Fg γ peptides inhibit ClfA binding to immobilized GST-Fg γ^1–17^ in solid phase assays. Wells were coated with 1 µg GST- Fg γ^1–17^ peptide. ClfA_229–545_ (100 nM) was pre-incubated with wild-type Fg γ^1–17^ peptide (WT γ^1–17^) or the P1 (G1A) to P17 (V17A) mutant peptide (50 µM) for 1 hr. (C) Binding of ClfA to immobilized GST-Fg γ^1–17^ and GST-Fg γ^1–17^_D16A_ using a solid-phase assay. Increasing concentrations of rClfA_229–545_ were incubated in microtiter wells containing 1 µg GST (circles), GST-Fg γ^1–17^ (triangles) or GST-Fg γ^1–17^_D16A_ (squares). Bound ClfA was detected with anti-His monoclonal antibodies as described in Material and Methods. (D) Binding of ClfA_229–545_ to Fg γ^1–17^ and Fg γ^1–17^_D16A_ peptides in solution using ITC.

In previous studies, a segment of ClfA composed of residues 221–559 was shown to bind to the C-terminal end of the human Fg γ-chain [10]. We designed, based on structural similarities with SdrG, a smaller ClfA construct (229–545) predicted to be composed only of the N2 N3 domains and showed that ClfA_229–545_ retained the Fg-binding activity. To identify specific residues in Fg that are important for binding to ClfA_229–545_, a panel of peptides (Fig. 1A) based on the Fg γ-chain sequence 395–411 (referred to as γ^1–17^) were synthesized in which each position was sequentially substituted with an alanine residue (alanines 11 and 14 were changed to serines). These peptides were tested as inhibitors in solid-phase binding assays, using a peptide concentration giving about 50% inhibition by the wild-type peptide. Peptides γ^1–17^_H6A_, γ^1–17^_H7A_, γ^1–17^_G10A_, γ^1–17^_Q13A_, γ^1–17^_A14S_ and γ^1–17^_G15A_ were significantly less potent inhibitors than the native sequence suggesting that the Fg residues H6, H7, G10, Q13, A14 and G15 interact with ClfA (Fig. 1B). Remarkably, peptides γ^1–17^_A11S_, γ^1–17^_D16A_ and γ^1–17^_V17A_ showed enhanced inhibition of ClfA binding to a recombinant form of residues 395–411 of the Fg γ-chain fused to a GST protein (GST-Fg γ_1–17_) compared to a peptide with the wild-type sequence, indicating a higher affinity of the peptide variants for ClfA.

The ability of ClfA_229–545_ to bind to the peptide containing the γ^1–17^_D16A_ mutation was further characterized. In solid-phase assays, ClfA binds to immobilized GST-Fg γ^1–17^ fusion protein with a lower affinity (K_d_ = 657 nM) compared to the mutated GST-Fg γ^1–17^_D16A_ (K_d_ = 35 nM) (Fig. 1C). In solution, using isothermal titration calorimetry (ITC) assays, (Fig. 1D), ClfA also binds with a lower affinity to the native γ^1–17^ peptide (K_d_ of 5.8 µM) compared to the mutant Fg γ^1–17^_D16A_ (K_d_ of 3 µM). Thus, although the apparent dissociation constants differ according to the assays used to estimate them, similar trends in affinity between the wild-type and the D16A mutation were observed.

Our results showed that alanine substitution at the C-terminal but not in the N-terminal region of the peptide affected MSCRAMM binding suggesting that the ClfA binding site is located at the very C-terminus of the Fg γ-chain (Fig. 1). Results also show that certain amino acid changes in the γ^1–17^ sequence enhance ClfA binding compared to the wild-type Fg sequence indicating that the human Fg γ C-terminal 17 residues may not be the optimum ligand for ClfA.

**Figure 2:**
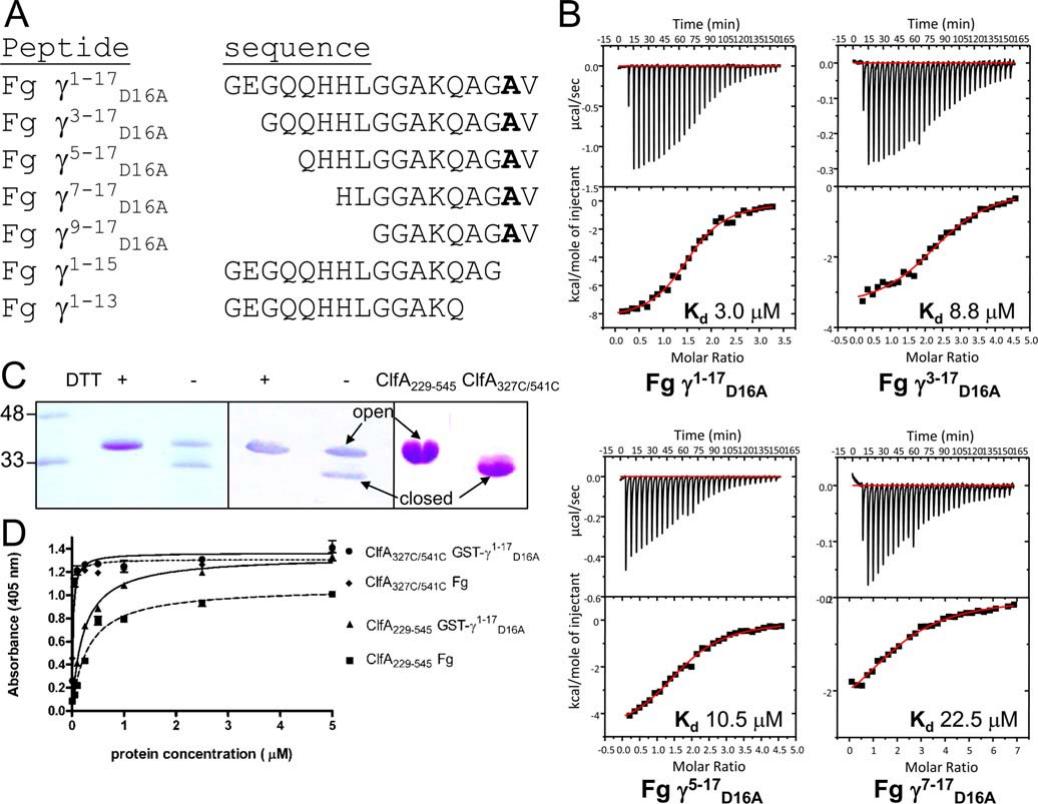
Fg and Fg γ^1–17^_D16A_ peptide truncations binding to different forms of ClfA. (A) Panel of Fg γ^1–17^_D16A_ peptides with N- and C-terminal truncations. (B) N-terminal deletions of Fg γ^1–17^_D16A_ peptide bind ClfA_229–545_ with decreasing affinities. N- and C-terminal truncated Fg γ^1–17^_D16A_ peptides were tested for their ability to bind ClfA_229–545_ in solution using ITC. (C) A stable closed conformation ClfA_229–545_ was engineered by introducing a disulfide bridge. The left panel shows a ligand blot of rClfA_D327C/K541C_. Recombinant proteins were run in an SDS-PAGE in the presence or absence of 5 mM DTT and stained with Coomassie Blue (left panel) or transferred to a PDVF membrane (middle panel). Transferred proteins were probed with Fg (10 mg/ml) and detected with anti-Fg and AP-conjugated secondary antibodies. (Right panel) The purified closed form of ClfA_327C/541C_ used for crystallization and ClfA229–545 were run in an SDS-PAGE and stained with Coomassie Blue (right panel). (D) The closed conformation of ClfA_D327C/K541C_ binds immobilized Fg and GST-Fg γ^1–17^_D16A_. ClfA_229–545_ or ClfA_D327C/K541C_ was incubated with wells coated with either Fg or GST-Fg γ^1–17^_D16A_ and detected with anti-His monoclonal antibodies as described in Materials and Methods.

Analysis of the previously solved SdrG-Fg peptide complex crystal structure showed that only 11 out of the 18 peptide residues interacted with the MSCRAMM. Similarly, only a part of the 17-residue γ-chain segment may be required for binding to ClfA. In order to establish the minimum Fg peptide required for binding to ClfA_229–545_, a series of N- and C-terminal truncations of the γ^1–17^_D16A_ peptide were synthesized (Fig. 2A). Truncations of 2, 4, 6 or 8 amino acids at the N-terminus of the Fg γ-peptide resulted in a reduced but detectable binding affinity when tested using ITC. There was a direct relationship between the length of the peptide and its affinity for ClfA. The smaller the peptide, the lower was the observed affinity for the MSCRAMM (Fig. 2B). Thus, the N-terminal residues of the Fg peptide (residues 1–8) are not critical for the interaction but may either contribute to or stabilize the binding of the peptide to ClfA. On the other hand, deletions of 2 or 4 residues from the C-terminal end of the γ^1–17^_D16A_ peptide abolished binding. These results indicate that the C-terminal amino acids of Fg are critical for binding to ClfA and are in agreement with a previous report that showed that Fg lacking the C-terminal residues AGDV in the γ chain (corresponding to residues 14–17 in the peptide) or a Fg-variant that replaces the last four γ-chain residues with 20 amino acids lacks the ability to bind recombinant ClfA_221–550_ and induce *S. aureus* clumping [10].

### A stabilized closed conformation of ClfA_229–545_ binds Fg with a higher affinity than the open form

The Fg binding mechanism of SdrG_276–596_ involves a transition from an open conformation, where the peptide binding trench between the N2 and N3 domains is exposed for ligand docking, to a closed conformation of the SdrG_276–596_ seen for the MSCRAMM in complex with the ligand peptide. The insertion of the N3 extension into the latching trench on N2, which represents the last step in the dynamic DLL binding mechanism, stabilizes the closed conformation of SdrG_237–596_ [31]. A closed conformation of apo SdrG N2N3, stabilized by introducing a disulfide bond between the end of the N3 latch and the “bottom” of N2, no longer binds Fg [31] demonstrating that for SdrG an open conformation is required for the initial docking of the ligand peptide. To explore if the binding of ClfA to Fg is also dependent on a movement of the latch we constructed a ClfA protein containing two cysteine substitutions. The locations of the cysteine mutations were determined using computer modeling and by sequence alignment to corresponding mutations in SdrG [31]. The mutant ClfA_D327C/K541C_ generated a stable, closed conformation form. This recombinant His-tag fusion protein was purified by Ni^+^ chelating chromatography; ion-exchange and gel permeation chromatography. The ClfA_D327C/K541C_ open and closed conformation forms were examined by SDS-PAGE analysis (Fig. 2C). Under non reducing conditions, the disulfide bonded closed form of ClfA_D327C/K541C_ migrated faster on SDS-PAGE than its non-disulfide bonded open form. Presumably, under non-reducing conditions, closed conformation mutants are more compact and migrate faster on SDS-PAGE than open conformation constructs. Under reducing conditions, the disulfide mutant and the wild-type protein migrate at the same rate. Surprisingly, the closed conformation of the disulfide mutant ClfA_D327C/K541C_ was able to bind Fg (Fig. 2C). Elisa-type binding assays where Fg or GST Fg γ^1–17^ peptide were coated in microtiter wells and incubated with ClfA showed that the closed conformation ClfA_D327C/K541C_ bound the ligand with a much lower apparent K_d_ (34 nM Fg; 20 nM GST-Fg γ^1–17^) compared to the wild-type ClfA_229–545_ (apparent K_d_ 305 nM Fg; 222 nM GST-Fg γ^1–17^) (Fig. 2D). These results demonstrate that an open conformation may not be required for Fg binding to ClfA and that Fg binding by ClfA involves a mechanism that is different from the DLL mechanism employed by SdrG.

### Crystal structure of ClfA_(229–545)D327C/K541C_ in complex with a 13 residue Fg-derived peptide

**Figure 3:**
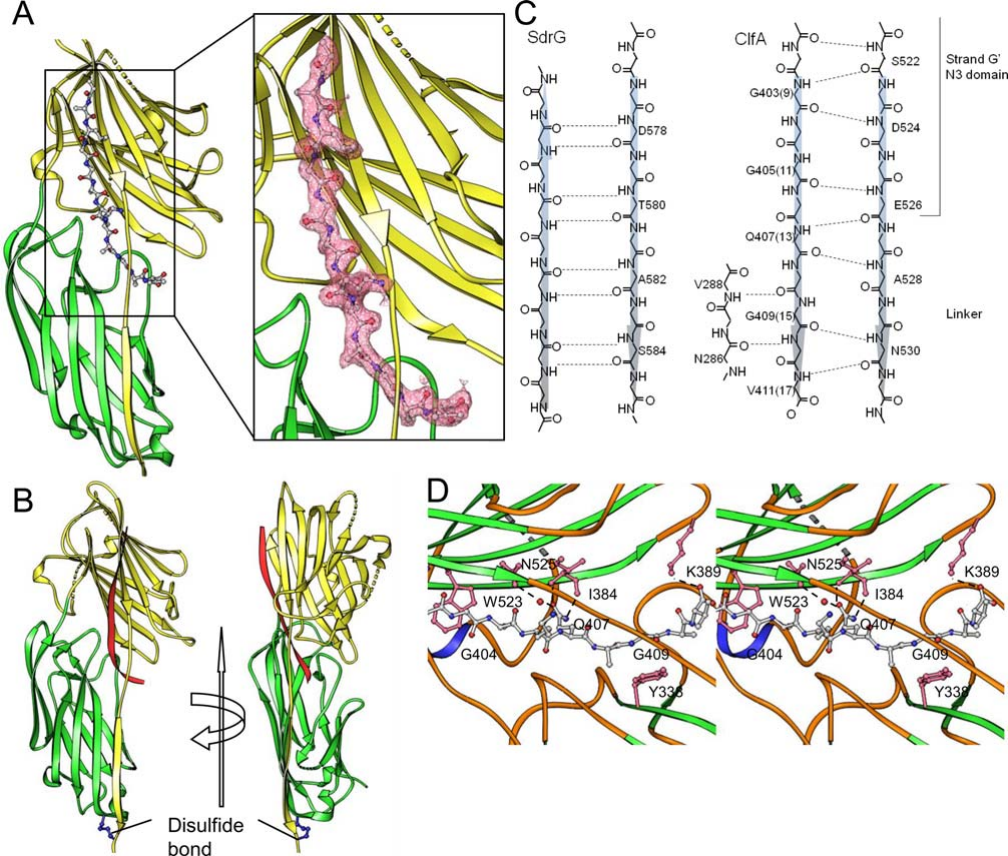
Representation of ClfA_D327C/K541C_ (N2–N3)-peptide complex. (A) Ribbon representation of ClfA-peptide (Fg γ-chain analog) complex. The peptide is shown as ball and stick model. 2Fo-Fc map around the peptide contoured at 1σ is shown in the close-up view. (B) Ribbon representation of ClfA_D327C/K541C_ –peptide complex in two orientations. The peptide molecule is shown in red and the engineered disulfide bond is shown as blue ball-and-stick object. (C) Schematic representation of ClfA-Fg γ-peptide main-chain parallel β-complementation interaction. The anti-parallel β-complementation observed in SdrG_273–597_-Fg β-peptide complex is also shown for comparison. The residue numbers of both the Fg γ-chain sequence and the peptide numbering (1–17), in parenthesis, are shown. (D) Stereo-view showing the side-chain interactions of the ClfA- Fg γ-peptide complex. Carbon atoms of the peptide are shown in grey; oxygen, red; nitrogen, blue. Side chain atoms of ClfA are shown as pink stick objects. Hydrogen bonds are shown as dotted lines.

Crystallization screens were carried out with ClfA_D327C/K541C_ in complex with several N-terminal truncations of the γ^1–17^_D16A_ peptide that were shown to bind ClfA. Crystals of the stable closed conformation of ClfA_229–545_ in complex with several peptides were obtained, but structure determination was attempted for only the ClfA_(229–545)D327C/K541C_-γ^5–17^_D16A_ peptide. The crystals of the ClfA-peptide complex diffracted to a 1.95 Å resolution. Two copies of the ClfA-peptide complex were found in the asymmetric part of the unit cell and are referred to as A∶C and B∶D. Although the 13 residue Fg γ^5–17^ chain synthetic peptide was used for crystallization, only 11 residues were identified completely in both copies of the complex. The two molecules of ClfA_D327C/K541C_ (A and B) are nearly identical with rms deviation of 0.3 Å for 312 Cα atoms and 0.55 Å for backbone atoms. As observed in the apo-ClfA_221–559_ structure [24], the ClfA_(229–545)D327C/K541C_ N2 and N3 domains adopt the DE-variant IgG fold. The overall structure of the ClfA_D327C/K541C_ peptide complex (A∶C) and the two different orientations of the complex are shown in Figure 3A and 3B respectively. The C-terminal extension of the N3 domain makes a β-sheet complementation with strand E of the N2 domain. This conformation is locked by the engineered disulfide bond as predicted by SDS-PAGE analysis (Fig. 2C) and confirmed by the crystal structure (Fig. S1). The two copies of the Fg γ-peptide molecules are nearly identical with rms deviation of 0.5 Å for 11 Cα atoms and 0.89 Å for backbone atoms. The interaction between the ClfA_D327C/K541C_ and the peptide buries a total surface area of 1849 Å^2^ and 1826 Å^2^ in the A∶C and B∶D complex, respectively. The interaction of the peptide with the N2 domain is predominantly hydrophobic in nature, in addition to a few main-chain hydrogen bonds (Fig. 3C). Interactions between the Fg peptide and the N3 domain are both hydrophobic and electrostatic with the electrostatic contribution coming almost entirely from the main chain-main chain hydrogen bonds due to the parallel β-sheet formation of the peptide with strand G of the N3 domain (Fig. 3C). The side-chain interactions between the peptide and ClfA are predominantly hydrophobic. The 11 C-terminal residues of the Fg γ-chain peptide sequence that interact with ClfA are composed of only two polar residues, Lys12 and Gln13. Side chain atoms of Lys12 point away and do not interact with the ClfA protein whereas Gln13 makes two hydrogen bonds with the main chain atoms of Ile384 in ClfA (Fig. 3D). A water-mediated interaction is also observed between Gln13 of the peptide and Asn525 of ClfA. Tyr338 in the N2 domain and Trp523 in the N3 domain play an important role in anchoring the peptide molecule. Tyr338 and Trp523 are stacked with residues Gly15 and Gly10, respectively. In addition, Met521 and Phe529 make hydrophobic interactions with Ala7 and Val17, respectively. The C-terminal residues of the peptide Ala14, Gly15, Ala16, and Val17 are buried between the N2–N3 domain interface with the terminal Val residue, presumably threaded through a preformed ligand binding tunnel after ClfA_D327C/K541C_ adopted its closed conformation. A hydrogen bond is observed between Lys389 of ClfA and the C-terminal carboxyl group of the peptide (Fig. 3D). Mutational studies showed that Tyr_338_Ala and Lys_389_Ala mutant ClfA showed significantly reduced binding to Fg [24] which corroborates with the structural results. Also an earlier study showed that E526A and V527S affected the binding [32]. The structure shows that these residues make main-chain interactions with the peptide (Fig. 3C). These residues are critical for the anchoring the peptide (Lock) and redirection of the latch.

### Structural differences between the closed conformation ClfA_D327C/K541C_-peptide complex and the apo-ClfA_221–559_ protein

**Figure 4:**
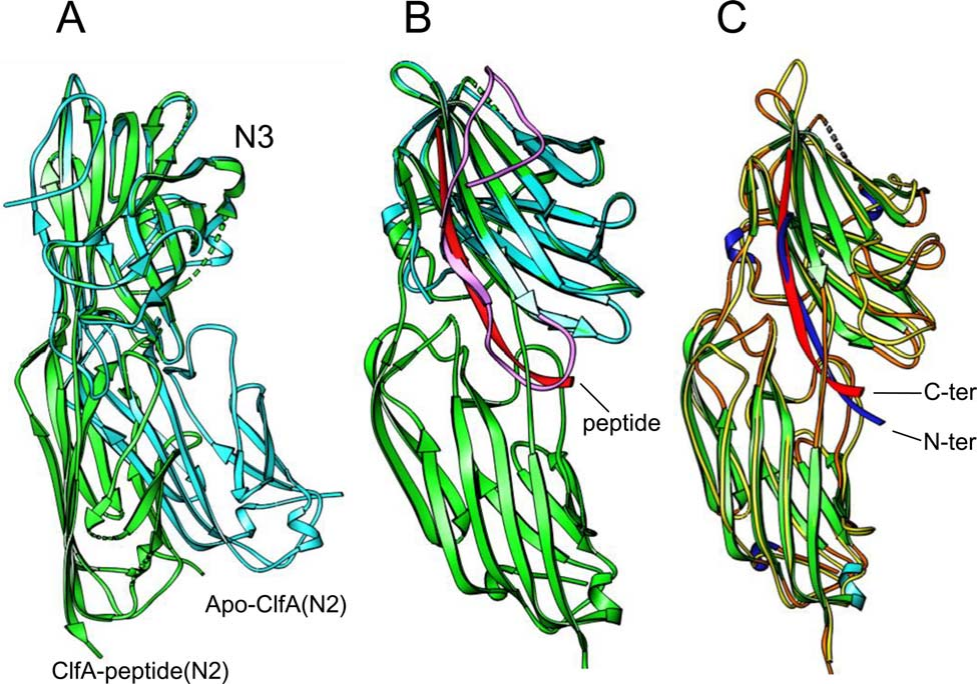
Superposition of apo-ClfA, ClfA-peptide and SdrG-peptide structures. (A) Superposition of apo-ClfA_221–559_, ClfA_D327C/K541C_ -peptide complex. The N3 domains of the two structures are superposed showing significant deviation in the inter-domain orientations. Apo-ClfA is shown as a cyan ribbon object and ClfA-peptide complex is shown in green. (B) Only N3 domain of apo-ClfA (cyan) is shown for clarity. The folded-back residues of the C-terminal residues of the apo-ClfA are shown in purple. The Fg γ-chain peptide is shown as red ribbon. (C) Superposition of ClfA-peptide and SdrG-peptide complexes. The peptide molecules corresponding to ClfA and SdrG complexes are shown as red and blue ribbon objects respectively. ClfA is colored by secondary structure and SdrG is shown as thin yellow uniform coil.

The individual N2 and N3 domains in the apo-ClfA_221–559_ and the closed form of ClfA_D327C/K541C_ are almost identical with rms deviations of 0.33 and 0.42 Å for molecule A and 0.35 and 0.42 Å for molecule B, but the relative orientation of the N2 and N3 domains are significantly different (Fig. 4A). This difference affects the association of the N2 and N3 domains. In the apo conformation, the buried surface area between the N2 and N3 domains is 87 Å^2^ compared to 367 Å^2^ in the closed form of the ClfA_(221–559)D327C/K541C_-peptide complex. In the apo-ClfA_221–559_, the C-terminal residues (Ala528-Glu559) of the N3 domain fold back and do not interact with the N2 domain. Moreover the folded-back segment completely occupies the binding site (Fig. 4B). Therefore, in the folded-back conformation, the ligand binding site appears not to be accessible to the peptide and thus this conformation appears to be inactive. It is presently unclear what the spatial arrangements of the N2N3 domains are in intact ClfA expressed on the surface of a staphylococcal cell. The two structures of these domains solved so far where one is active and the other inactive form suggests a possible regulation of ClfA's Fg binding activity by external factors. One such factor may be Ca^2+^ which has been shown to inhibit ClfA-Fg binding [32]. Alternatively, it is possible that the folded-back conformation (which is a larger protein construct) is only one of the many possible conformations adopted by the unbound protein. Molecular modeling shows that the two domains in the folded-back conformation could adopt an orientation similar to their orientation in the ClfA-peptide complex (Fig. S2). Most likely, the structural rearrangements responsible for the transition of ClfA from an open unbound to the closed bound form are complex and involve different intermediate forms.

### Structural similarities/differences between the closed form of the ClfA-peptide and SdrG-peptide complexes

The major difference between Fg-binding to ClfA and SdrG is that the directionality of the bound ligand peptide is reversed (Fig. 4C). The C-terminal residues of the ligand is docked between the N2 and N3 in ClfA and makes a parallel β-sheet complementation with strand G of the N3 domain, whereas in SdrG, the N-terminal residues of the ligand are docked between the N2 and N3 domains and form an anti-parallel β-sheet with the G strand. In both cases there are 11 ligand residues that make extensive contact with the MSCRAMM but with one residue shifted towards the N3 domain in ClfA. Of these 11 residues, 7 and 11 residues participate in the β-strand complementation of SdrG and ClfA, respectively. Although the peptide binding model of ClfA is different to that of SdrG, the inter-domain orientations of the two MSCRAMMS are very similar [25]. Superposition of 302 corresponding atoms in the N2 and N3 domains of ClfA and SdrG showed a small rms deviation of 0.65 Å indicating the high structural similarity between the two MSCRAMMS. Another striking difference is that ClfA does not require an open-conformation for ligand binding, whereas Fg can not bind to a stabilized closed conformation of SdrG. ClfA binds the C-terminal end of Fg and the last few residues of the γ-chain presumably can be threaded in to the binding pocket. In the SdrG-Fg interaction, the binding segment in Fg does not involve the seven N-terminal residues of the ligand and therefore an open conformation may be required for ligand binding.

### Comparison of Fg binding to ClfA and the platelet integrin α_IIb_β_3_

**Figure 5:**
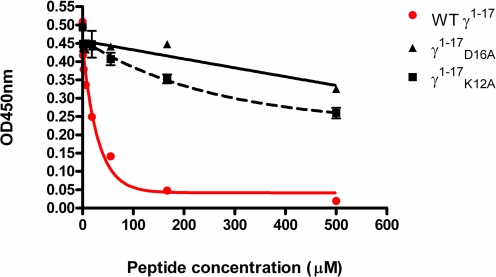
The γ^1–17^_D16A_ and γ^1–17^_K12A_ peptides bind weakly to platelet integrin α_IIb_β_3_. Inhibition of Fg γ peptides (γ^1–17^_D16A_, γ^1–17^_K12A_ and WT γ^1–17^) on binding of full length Fg to immobilized α_IIb_β_3_. Wild-type Fg-γ^1–17^ peptide (circle) inhibits Fg binding to α_IIb_β_3_ whereas γ^1–17^_D16A_ (triangle) and γ^1–17^_K12A_ (square) peptides have very little inhibitory effect. Various concentrations of peptides were mixed with a fixed Fg concentration (10 nM) and the mixture added to immobilized α_IIb_β_3_. Bound Fg was detected using antibodies against human Fg as described in Materials and Methods.

The C-terminus of Fg γ-chain, which is targeted by ClfA, is also recognized by the α_IIb_β_3_ integrin in Fg induced platelet aggregation, a vital step in thrombosis [10],[33]. The Fg γ-chain complex with α_IIb_β_3_ structure is not available but structures of related complexes provide clues on how α_IIb_β_3_ likely interact with Fg [34]. In addition, the crystal structure of the α_v_β_3_ integrin in complex with an RGD ligand provided a structural model of a similar ligand-integrin interaction [35]. In this structure, the Asp (D) residue of the RGD sequence coordinates with the metal ion in the Metal Ion Dependent Adhesion Site (MIDAS) of the integrin and thus plays a key role in the interaction. The platelet specific integrin α_IIb_β_3_ recognizes ligands with an RGD sequence or the sequence Lys-Gln-Ala-Gly-Asp-Val found in Fg [34]. Structural studies with drug molecules that antagonize the integrin-RGD or -Fg interaction showed that each of the drug molecules contains a carboxyl group moiety that mimics the aspartic acid and a basic group that mimics the Arg (or Lys in the case of Fg) in the ligand [34]. These results suggest that the Lys and Asp residues in the C-terminal γ-chain sequence are critical for the interaction with integrin. Interestingly, our studies have shown that these Lys and Asp residues in Fg are not critical for ClfA binding (Fig. 1B). In fact, substitution of Asp with Ala (γ^1–17^_D16A_) results in a higher binding affinity. Absence of a strong interaction with Lys12 in the ClfA-peptide complex structure also correlates with the biochemical data, suggesting that Arg is not a key player in the ClfA-Fg interaction. In general, our studies show that K406 and D410, which are essential for the platelet integrin α_IIb_β_3_-Fg interaction, are dispensable for the ClfA-Fg interaction. To experimentally examine this proposed difference, the ability of the synthesized Fg WT γ^1–17^ and mutated peptides (γ^1–17^_D16A_ and γ^1–17^_K12A_) to inhibit full length Fg binding to α_IIb_β_3_ was analyzed by an inhibitory ELISA type assay (Fig. 5). The WT γ^1–17^ peptide completely inhibited the binding of full-length fibrinogen to α_IIb_β_3_ whereas γ^1–17^_D16A_ and γ^1–17^_K12A_ weakly inhibited Fg binding to α_IIb_β_3_. These results clearly demonstrated that the γ^1–17^_D16A_ and γ^1–17^_K12A_ peptides bind weakly to platelet integrin and therefore could serve as specific antagonists of Fg-ClfA interaction.

### Conclusions

Based on the results presented here, we postulate that the mechanism of interaction between ClfA and Fg is a variation of the “Dock, Lock and Latch (DLL)” model of SdrG binding to Fg. In the DLL model of binding, the apo-form of the SdrG is in an open conformation to allow the ligand access to the binding cleft. A closed conformation of SdrG is unable to bind Fg. In the ClfA model, we believe that the peptide may thread into the cavity formed in a stabilized closed configuration and therefore the ClfA-Fg binding mechanism could be called “Latch and Dock”.

In the case of CNA, a collagen binding MSCRAMM from *S. aureus*, the collagen molecule binds to CNA through a “collagen hug” model [36] which represents yet another variant of the DLL binding mechanism. All three MSCRAMM-ligand structures determined so far, SdrG, CNA and the ClfA have different ligand binding characteristics and mechanisms, although the overall structures of the ligand binding regions of these MSCRAMMs are very similar. These observations suggest that an ancestral MSCRAMM has evolved along different paths to accommodate different ligands without greatly altering the overall organization of the proteins.

The co-crystal structure of ClfA in complex with the C-terminal region of the γ-chain of Fg will allow the design of potent antagonist of the ClfA-Fg interaction. The Fg based peptide analogs that antagonize the ClfA-Fg interaction but not affect the α_IIb_β_3_ integrin interaction could serve as a starting point to develop novel anti-staphylococcal therapeutic agents that do not affect the α_IIb_β_3_.

## Materials and Methods

### Bacterial Strains, Plasmids, and Culture Conditions

*Escherichia coli* XL-1 Blue (Stratagene) was used as the host for plasmid cloning and protein expression. Chromosomal DNA from *S. aureus* strain Newman was used to amplify the ClfA DNA sequence. All *E. coli* strains containing plasmids were grown on LB media with ampicillin (100 µg/ml).

### Manipulation of DNA

DNA restriction enzymes were used according to the manufacturer's protocols (New England Biolabs) and DNA manipulations were performed using standard procedures [37]. Plasmid DNA used for cloning and sequencing was purified using the Qiagen Miniprep kit (Qiagen). DNA was sequenced by the dideoxy chain termination method with an ABI 373A DNA Sequencer (Perkin Elmer, Applied Biosystems Division). DNA containing the N-terminal ClfA sequences were amplified by PCR (Applied Biosystems) using Newman strain chromosomal DNA as previously described [38]. The synthetic oligonucleotides (IDT) used for amplifying *clfA* gene products are listed in Table S1.

### Construction of disulfide mutants

Cysteine mutations were predicted by comparing ClfA_221–559_ to SdrG_(273–597)_ disulfide mutant with stable closed conformations [31] and by computer modeling. A model of ClfA in closed conformation was built based on the closed conformation of the SdrG-peptide complex [25]. The Cβ-Cβ distances were calculated for a few residues at the C-terminal end of the latch and strand E in the N2 domain. Residue pairs with Cβ-Cβ distance less than 3 Å were changed to cysteines to identify residues that could form optimum disulfide bond geometry. The D327C/K541C mutant was found to form a disulfide bond at the end of the latch. The cysteine mutations in ClfA_D327C/K541C_ were generated by overlap PCR [39],[40]. The forward primer for PCR extension contained a *Bam*HI restriction site and the reverse primer contained a *Kpn*I restriction site. The mutagenesis primers contained complementary overlapping sequences. The final PCR product was digested with *Bam*HI and *Kpn*I and was ligated into same site in the expression vector pQE-30 (Qiagen). All mutations were confirmed by sequencing. The primers used are listed in Table S1.

### Expression and Purification of Recombinant Proteins

*E. coli* lysates containing recombinant ClfA and GST-Fg γ-chain fusion proteins were purified as previously described [32]. PCR products were subcloned into expression vector pQE-30 (Qiagen) to generate recombinant proteins containing an N-terminal histidine (His) tag as previously described [10]. The recombinant ClfA His-tag fusion proteins were purified by metal chelation chromatography and anion exchange chromatography as previously described [23]. To generate recombinant ClfA_229–545_ and ClfA_221–559_ proteins, PCR-amplified fragments were digested with *Bam*HI and *Kpn*I and cloned into *Bam*HI/*Kpn*I digested pQE-30. The primers used to generate the recombinant constructs are listed in Table S1. The reactions contained 50 ng of strain Newman DNA, 100 pmol of each forward and reverse primers, 250 nM of each dNTP, 2 units of *Pfu* DNA polymerase (Stratagene) and 5 µl *Pfu* buffer in a total volume of 50 µl. The DNA was amplified at 94°C for 1 min, 48°C for 45 sec; 72°C for 2 min for 30 cycles, followed by 72°C for 10 min. The PCR products were analyzed by agarose gel electrophoresis using standard methods [37] and purified as described above.

### Enzyme-linked Immunosorbent Assay

The ability of the wild-type ClfA_229–545_ and disulfide ClfA mutants to bind Fg was analyzed by ELISA-type binding assays. Immulon 4HBX Microtiter plates (Thermo) were coated with human Fg (1 µg/well) in HBS (10 mM HEPES, 100 mM NaCl, 3 mM EDTA, pH 7.4) over-night at 4°C. The wells were washed with HBS containing 0.05% (w/v) Tween-20 (HBST) and blocked with 5% (w/v) BSA in HBS for 1 h at 25°C. The wells were washed 3 times with HBST and recombinant ClfA proteins in HBS were added and the plates were incubated at 25°C for 1 h. After incubation, the plates were washed 3 times with HBST. Anti-His antibodies (GE Healthcare) were added (1∶3000 in HBS) and the plates were incubated at 25°C for 1 h. The wells were subsequently washed 3 times with HBST and incubated with goat anti-mouse-AP secondary antibodies (diluted 1∶3000 in HBS; Bio-Rad) at 25°C for 1 h. The wells were washed 3 times with HBST and AP-conjugated polyclonal antibodies were detected by addition of *p*-nitrophenyl phosphate (Sigma) in 1 M diethanolamine (0.5 mM MgCl_2_, pH 9.8) and incubated at 25°C for 30–60 min. The plates were read at 405 nm in a ELISA plate reader (Thermomax, Molecular Devices). For the inhibition assays, recombinant ClfA_229–545_ was pre-incubated with Fg γ peptides in HBS for 1 h at 37°C. The recombinant protein-peptide solutions were then added to plates coated with 1 µg/well GST fusion protein containing the native human Fg γ 395–411 sequence (called GST-Fg γ^1–17^) and bound protein was detected as described above. If the peptide binds ClfA it would inhibit binding of the GST-Fg γ^1–17^ to the MSCRAMM.

For α_IIb_β_3_ inhibition assay, Immulon 4HBX Microtiter 96-well plates (Thermo) were coated with α_IIb_β_3_ (0.25 µg/well) in TBS (25 mM Tris, 3 mM KCl, 140 mM NaCl, pH 7.4) over night at 4°C. The wells were washed with TBS containing 0.05% (w/v) Tween-20 (TBST). After blocking with 3% (w/v) BSA dissolved in TBS for 1 h at RT, 10 nM of full length Fg was applied in the presence of either WT γ^1–17^, γ^1–17^_D16A_ or γ^1–17^_K12A_ peptides and plates were incubated at RT for another hour. The bound full length Fg was then detected by goat anti human Fg (1∶1000 dilution, Sigma) antibody followed by horseradish peroxidase-conjugated rabbit anti-goat IgG antibody (1∶1000 dilution, Cappel). After incubation with 0.4 mg/ml of substrate, *o*-phenylenediamine dihydrochloride (OPD, Sigma) dissolved in phosphate-citrate buffer, pH 5.0, bound antibodies were determined in an ELISA reader at 450 nm. The proteins, antibodies and peptides were diluted in TBST containing 1% (w/v) BSA, 2 mM MgCl_2_, 1 mM of CaCl_2_ and MnCl_2_.

### Synthesis of γ-chain Peptides

The wild-type and mutated peptides corresponding to the 17 C-terminal residues of the fibrinogen γ-chain (395–411) and truncated versions of this peptide (listed in Figure 2A) were synthesized as previously described and purified using HPLC [10].

### Isothermal Titration Calorimetry

The interaction between ClfA proteins and soluble Fg peptides was analyzed by Isothermal titration calorimetry (ITC) using a VP-ITC microcalorimeter (MicroCal). The cell contained 30 µM ClfA and the syringe contained 500–600 µM peptide in HBS buffer (10 mM HEPES, 150 mM NaCl, pH 7.4). All samples were degassed for 5 min. The titration was performed at 30°C using a preliminary injection of 5 µl followed by 30 injections of 10 µl with an injection speed of 0.5 µl/sec. The stirring speed was 300 rpm. Data were fitted to a single binding site model and analyzed using Origin version 5 (MicroCal) software.

### Crystallization

The ClfA_D327C/K541C_ protein was purified as described earlier and concentrated to 30 mg/ml. The synthetic γ-chain peptide analogs, P16 and N-terminal truncations of P16 (P16 -2Nt, P16 -4Nt and P16 -6Nt) were mixed with the protein at 1∶20 molar ratio and left for 30 min at 5° C. This mixture was screened for crystallization conditions. Small needles of the ClfA/P16 -2Nt, -4Nt and -6Nt were obtained during initial search of the crystallization condition, but we could only successfully optimize ClfA/P16 -4Nt and ClfA/P16 -6Nt. Diffraction quality crystals were obtained by mixing 2 µl of protein solution with 2 µl of reservoir solution containing 16–20% PEG 8K, 100 mM succinic acid pH 6.0.

### X-ray data collection, Structure Solution and refinement

Crystals of ClfA/ P16 -4Nt were flash frozen with a stabilizing solution containing 20% glycerol. Diffraction data were measured on Rigaku R-Axis IV^++^ detector. A total of 180 frames were collected at a detector distance of 120 mm with 1° oscillation. Data were indexed, integrated and scaled using d*terk [41]. The crystals diffracted to 1.95 Å and the data statistics were listed in Table 1. Calculation of the Matthews coefficient suggested the presence of 2 copies of the molecule in the unit cell of the triclinic cell. The structure was solved by molecular replacement (MR) with the program PHASER [42] using individual N2 and N3 domains of ClfA as search model. Solutions for the N3 domain were obtained for the two copies followed by the solutions of N2 domains. Data covering 2.5–15 Å were used for the molecular replacement solution. Electron density maps calculated during the initial rounds of refinement showed interpretable density for 11 out of 13 peptide residues in both the copies of the complex. Modeling building of the peptide and rebuilding of a few loop regions were performed using the program COOT [43]. A few cycles of ARP/WARP [44] were performed to improve the map and for the building of water model. After a few cycles of refinement using Refmac5.2 [45], electron density was clear for only the backbone atoms for two remaining N-terminal residues of the peptide molecule D and one residue for peptide C. The final model of ClfA included residues 230–299, 303–452, 456–476 and 479–545 in molecule A and 230–438, 440–476 and 479–542 in molecule B. The structure was refined to a final R-factor of 21.1% and R-free of 27.9%. Stereochemical quality of the model was validated using PROCHECK [46]. Molecular modeling studies were performed using InsightII software (Accrelys Inc). Figures were made using RIBBONS [47]. The atomic coordinates and structure factors of the complex structure have been deposited in Protein data bank with accession number; 2vr3.

**Table 1:** Crystallographic data measurement and refinement data.

Cell dimensions	
a, b, c (Å)	35.43, 61.84, 81.78
α, β, γ (°)	85.44, 81.84, 82.45
Space group	P1
Resolution (Å)	1.95–15.0
Reflections total/unique	86051/46090
Completeness (%)	93.9
R_merge_ *	0.074
Number of molecules in the asymmetric unit	2
Rfactor/ R_free_ +	0.211/0.279
Bond rms deviation (Å)	0.015
Angle rms deviation (°)	1.64
Average B value (Å)	29.9
No of non-hydrogen atoms	5226
Protein	4558
Peptide	141
Water	527
Rms deviations from ideal values	
Bond lengths (Å)	0.22
Bond Angles (°)	1.95
PDB ID	2vr3

*: R_merge_ = Σ|I_j_−〈I〉|/Σ I_i_; where I_j_ is the measured and 〈I〉 is the mean intensity of reflection hkl.

**+:** R_free_ is calculated over 2% of randomly selected reflections not included in the refinement.

## Supporting Information

Figure S1 Stereo view showing the disulfide bond in the ClfAD327C/K541C. 2Fo-Fc map around the Cys327 and Cys541 contoured at 1σ is shown. Carbon atoms are colored grey and the sulfur atoms in yellow.
(5.03 MB TIF)

Figure S2 Ribbon representation of modeled ClfA229–559 in ligand bound N2–N3 orientation. Residues that make clashes are shown as stick objects. This model was built to understand if the altered N2–N3 orientation of the apo-form of ClfA (Fig. 4A) is due to the folded-back conformation, a model of the apo-ClfA221–559 was constructed with the folded-back N3 domain and the N2 domain adopting an N2–N3 orientation similar to that observed in the closed form of the ClfA-peptide complex. This model shows that Tyr338 in the N2 domain makes severe clashes with residues Ser535 and Gly534 of the folded back segment. An alternate conformation for these residues is unlikely due to spatial constraints. Additional clashes were also observed between Ala 254 and Gly 536. Thus, it is unlikely that the two domains in the folded-back conformation could adopt an orientation similar to their orientation in the ClfA-peptide complex.
(9.32 MB TIF)

Table S1 (0.02 MB DOC)
