# Supplementary figures and images for: A Structural Model of the Staphylococcus aureus ClfA–Fibrinogen Interaction Opens New Avenues for the Design of Anti-Staphylococcal Therapeutics
Source: PLoS Pathog. 2008 Nov 28;4(11):e1000226. doi: 10.1371/journal.ppat.1000226 (PMC2582960; doi:10.1371/journal.ppat.1000226)

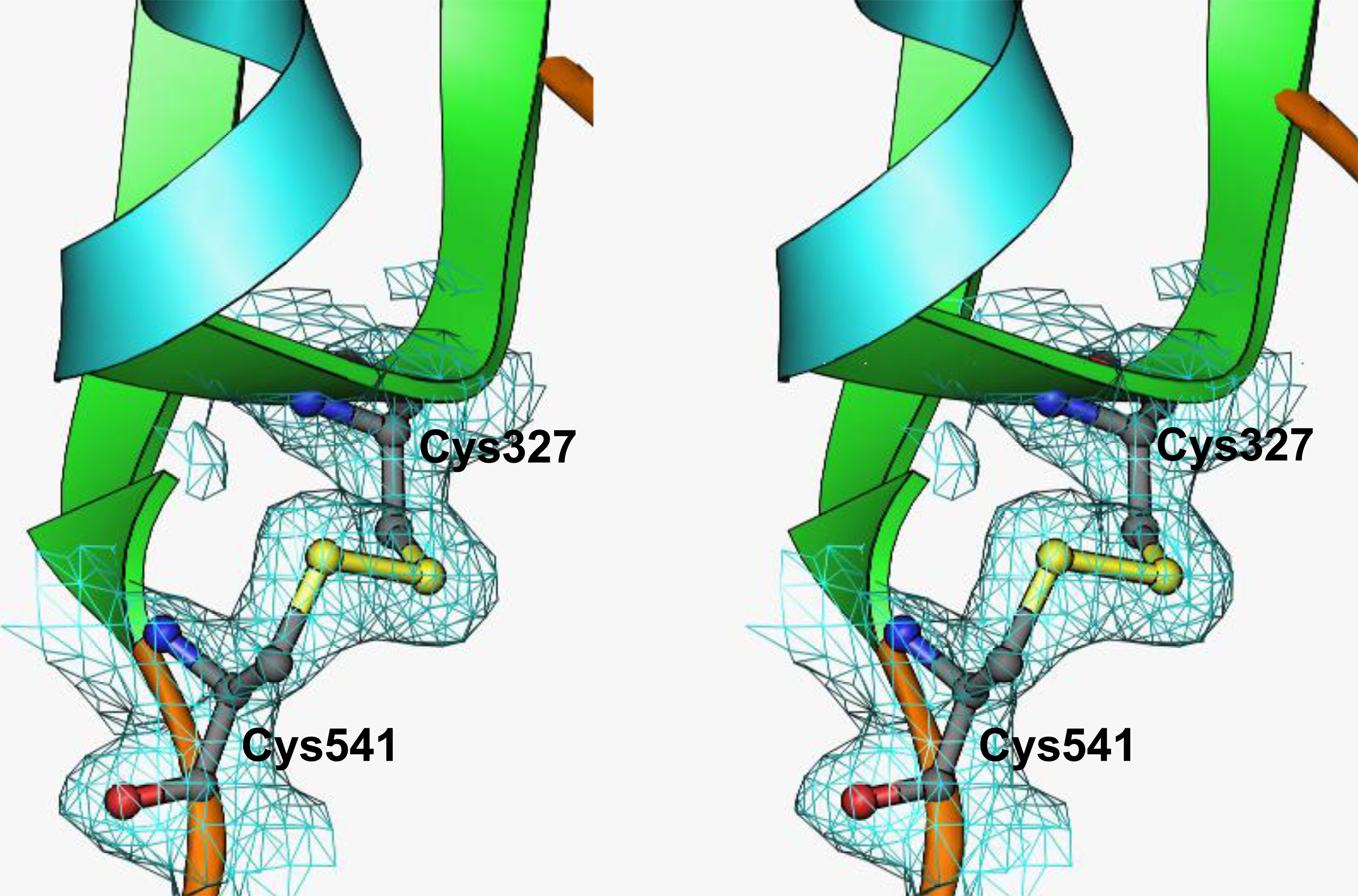

Supplement: Figure S1 — Stereo view showing the disulfide bond in the ClfAD327C/K541C. 2Fo-Fc map around the Cys327 and Cys541 contoured at 1σ is shown. Carbon atoms are colored grey and the sulfur atoms in yellow. (5.03 MB TIF) [file ppat.1000226.s001.tif]

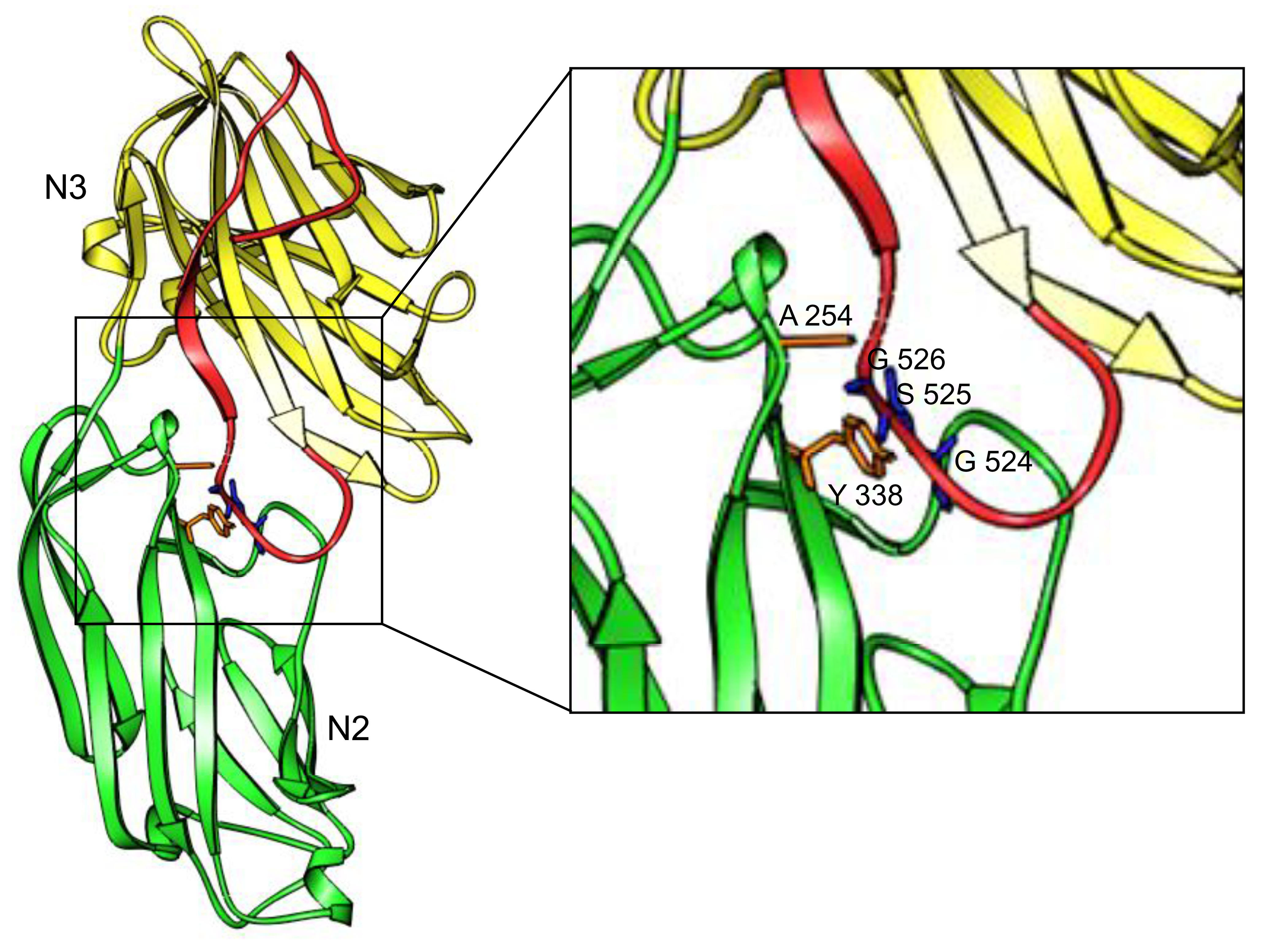

Supplement: Figure S2 — Ribbon representation of modeled ClfA229–559 in ligand bound N2–N3 orientation. Residues that make clashes are shown as stick objects. This model was built to understand if the altered N2–N3 orientation of the apo-form of ClfA (Fig. 4A) is due to the folded-back conformation, a model of the apo-ClfA221–559 was constructed with the folded-back N3 domain and the N2 domain adopting an N2–N3 orientation similar to that observed in the closed form of the ClfA-peptide complex. This model shows that Tyr338 in the N2 domain makes severe clashes with residues Ser535 and Gly534 of the folded back segment. An alternate conformation for these residues is unlikely due to spatial constraints. Additional clashes were also observed between Ala 254 and Gly 536. Thus, it is unlikely that the two domains in the folded-back conformation could adopt an orientation similar to their orientation in the ClfA-peptide complex. (9.32 MB TIF) [file ppat.1000226.s002.tif]
