# Supplementary material for: A Structural Model of the Staphylococcus aureus ClfA–Fibrinogen Interaction Opens New Avenues for the Design of Anti-Staphylococcal Therapeutics
Source: PLoS Pathog. 2008 Nov 28;4(11):e1000226. doi: 10.1371/journal.ppat.1000226 (PMC2582960; doi:10.1371/journal.ppat.1000226)
Supplement: Table S1 — (0.02 MB DOC) [file ppat.1000226.s003.doc]

Supplementary Table S1

ClfA229 5’-CCCGGATCCGGCACAGATATTACGAAT-3’

ClfA545 5’-CCCGGTACCTCAAGGAACAACTGGTTTATC-3’

For disulfide mutant:

rClfA327 5’-TGCTTTTACATCACATTTAGTATTTAC-3’

fClfA327 5’-GTAAATACTAAATGTGATGTAAAAGCA-3’

ClfA541 5’-CCCGGTACCTCAAGGAACAACTGGACAATCGATACCGTC-3’
